# Altered structural and effective connectivity in anorexia and bulimia nervosa in circuits that regulate energy and reward homeostasis

**DOI:** 10.1038/tp.2016.199

**Published:** 2016-11-01

**Authors:** G K W Frank, M E Shott, J Riederer, T L Pryor

**Affiliations:** 1 Department of Psychiatry, University of Colorado School of Medicine, University of Colorado Anschutz Medical Campus, Aurora, CO, USA; 2 Neuroscience Program, University of Colorado Denver, Anschutz Medical Campus, Aurora, CO, USA; 3 Eating Disorders Center Denver, Denver, CO, USA

## Abstract

Anorexia and bulimia nervosa are severe eating disorders that share many behaviors. Structural and functional brain circuits could provide biological links that those disorders have in common. We recruited 77 young adult women, 26 healthy controls, 26 women with anorexia and 25 women with bulimia nervosa. Probabilistic tractography was used to map white matter connectivity strength across taste and food intake regulating brain circuits. An independent multisample greedy equivalence search algorithm tested effective connectivity between those regions during sucrose tasting. Anorexia and bulimia nervosa had greater structural connectivity in pathways between insula, orbitofrontal cortex and ventral striatum, but lower connectivity from orbitofrontal cortex and amygdala to the hypothalamus (*P<*0.05, corrected for comorbidity, medication and multiple comparisons). Functionally, in controls the hypothalamus drove ventral striatal activity, but in anorexia and bulimia nervosa effective connectivity was directed from anterior cingulate via ventral striatum to the hypothalamus. Across all groups, sweetness perception was predicted by connectivity strength in pathways connecting to the middle orbitofrontal cortex. This study provides evidence that white matter structural as well as effective connectivity within the energy-homeostasis and food reward-regulating circuitry is fundamentally different in anorexia and bulimia nervosa compared with that in controls. In eating disorders, anterior cingulate cognitive–emotional top down control could affect food reward and eating drive, override hypothalamic inputs to the ventral striatum and enable prolonged food restriction.

## Introduction

Anorexia and bulimia nervosa are severe psychiatric disorders with high mortality.^1^ Although anorexia nervosa is mainly characterized by severe underweight and bulimia nervosa individuals are at normal to high weight and regularly binge and purge,^2^ there are many overlapping symptoms, such as food restriction, excessive exercise, altered interoceptive perception including hunger and appetite^3^ as well drive for thinness and body dissatisfaction. Anorexia and bulimia nervosa aggregate in families, and shared biological underpinnings have been hypothesized.^4^ The brain circuitry of food intake regulation depends on the interaction of the hypothalamus, which has a central role in energy homeostasis, with brain regions such as prefrontal and orbitofrontal cortex (OFC), insula, midbrain and ventral striatum, a brain circuitry that integrates taste perception, food reward value and cognitive–emotional associations with food.^5^ Alterations in those circuits in both disorders could point toward shared vulnerabilities.

Brain research in eating disorders has started to shed light on how altered brain structure or function may be common to anorexia and bulimia nervosa. Positron emission tomography studies suggested in both disorders increased serotonin 1A as well as cannabinoid type 1 receptors,^6, 7^ neurotransmitter receptors associated with food intake modulation and sweet taste perception.^8, 9, 10^ Structural brain imaging studies have been inconsistent. One study that directly compared the two disorders in a nutritionally highly controlled environment found larger OFC volumes in anorexia and bulimia nervosa^11^—a brain region that processes food pleasantness and regulates food intake.^12^ Others found increased somatosensory cortex volumes in both disorders.^13^ Functional magnetic resonance brain imaging (fMRI) indicated greater insula activation to food images,^14^ as well as higher resting-state synchrony between anterior cingulate cortex and precuneus,^15^ although other studies found opposite brain activation in response to visual food cues^16^ or sweet taste stimulation.^17, 18, 19^ Studies that focused on brain white matter found reduced white matter integrity (fractional anisotropy, FA) in anorexia and bulimia nervosa within the fornix.^20, 21, 22^

Research has helped to better understand how brain networks are structurally or functionally connected.^23^ One method is to investigate how strongly white matter tracts connect those brain regions, expressed as streamlines as an indicator of fiber amount.^24^ Another method is to investigate how brain regions functionally interact; this so-called dynamic causal or effective connectivity provides indication that brain region drives activation in another.^25, 26^ A few studies exist in eating disorders that have investigated effective connectivity, but not with respect to taste processing, which is the focus of our work. One study using resting-state data and Granger causality found that individuals with anorexia nervosa had higher effective connectivity from medial OFC and insula to inferior frontal gyrus, but lower effective connectivity from the frontal gyrus to the cingulate cortex.^27^ One small study suggested connectivity differences between anorexia nervosa and control groups between medial occipital cortex, extrastriate and fusiform body areas in response to viewing pictures of bodies or chairs.^28^ A study where individuals with anorexia, bulimia nervosa and controls viewed images of food and non-food items found no effective connectivity differences between insula, orbitofrontal and frontal cortex between anorexia nervosa and controls, but the bulimia nervosa group did not have connectivity between left insula and right frontal cortex that the other study groups had.^14^ A very recent report studied connectivity between the nucleus accumbens and the OFC using resting-state images in anorexia nervosa at two time points, before and after weight restoration.^29^ There the anorexia nervosa group showed an effective connectivity direction from the OFC to the nucleus accumbens, as well as greater anatomical connectivity strength using probabilistic tractography between the two regions of interest. One study from our group in individuals long-term recovered from anorexia nervosa found greater white matter connectivity strength from the insula to ventral striatum and OFC.^30^ Interestingly, duration of illness positively predicted connectivity strength of those tracts, suggesting a process compensating for effects from the illness and maybe affecting food reward-circuit function. In summary, few studies are available on effective or structural connectivity in eating disorders, and the varying methods and results do not provide a uniform model of brain function. One question that has to be raised in this context is what type of task is most suitable to elicit effective connectivity. We chose taste processing because we have a good understanding of the taste and taste-reward circuitry in the brain. Here we wanted to study this circuitry across multiple regions for a comprehensive assessment of structural and effective connectivity.

In this study we used a multimodal imaging approach to test the hypothesis that individuals ill with anorexia and bulimia nervosa have greater white matter connectivity across the energy homeostasis and cognitive-emotional reward circuitry. Furthermore, we wanted to study how regional activation during taste of sucrose solution is coupled, that is, what the pattern of effective connectivity or neural information flow is within this circuitry. We expected that we would find in the eating-disorder groups' indication that cognitive control regions would influence subcortical appetite and taste reward function, suggesting a top–down control mechanism.^31^ In contrast, we expected that in controls hypothalamic signals would rather influence reward system activation, presumably transmitting energy balance information to drive the food approach.^32^

## Materials and methods

### Participants

Twenty-six women with restricting-type anorexia nervosa, 25 with bulimia nervosa, as well as 26 healthy comparison women participated in the study. The sample size was based on our previous study that indicated adequate power for this type of study and analysis.^30^ Participants in the eating-disorder groups were recruited from Children's Hospital Colorado or Eating Disorders Center Denver. Eating disorder subjects were within 1**–**2 weeks of program-prescribed food intake to avoid acute effects of starvation and dehydration. Healthy comparison women were recruited through local advertisements. The Structured Clinical Interview for Diagnostic and Statistical Manual of Mental Disorders, 4th Edition diagnoses was administered by a doctoral-level interviewer. All participants were right-handed, without history of head trauma, neurological disease, major medical illness, psychosis or substance-use disorders. The study was approved by the Colorado Multiple Institutional Review Board, and all participants provided written informed consent.

### Behavior assessments

Study participants completed the Eating Disorder Inventory-3 (EDI-3),^33^ Temperament and Character Inventory (TCI),^34^ Spielberger State and Trait Anxiety Inventory (STAI),^35^ Beck Depression Inventory (BDI)^36^ and Revised Sensitivity to Punishment and Reward Questionnaire (SPSRQ).^37^ Before brain-imaging subjects rated sucrose sweetness on a 9-point likert scale, 1=dislike very much to 9=like very much.

### Brain imaging procedures

Before brain imaging between 0800 and 0900 hours, eating-disorder individuals ate their meal plan breakfast; controls had a breakfast matched in quality and calories to the average meal plan breakfast. Brain images were acquired on a GE Signa 3T scanner: (1) diffusion-weighted imaging (DWI) included 25 DWI diffusion directions and one T2-weighted (*b*=0) baseline image; 45 slices per image in anterior–posterior commissure orientation (128x128 matrix, repetition time (TR)/echo time (TE)=16 000/82.6 ms, field of view=26 cm, *b*-value=1000, ASSET, slice thickness/gap=2.6/0 mm). (2) fMRI T2* weighted echo-planar imaging for blood oxygen-dependent functional activity was performed, voxel size 3.4 × 3.4 × 2.6 mm, TR 2100 ms, TE 30 ms, angle 70°, 30 slices, interleaved acquisition and 2.6 mm slice thickness with 1.4 mm gap.

### fMRI task

We adapted the design used by O'Doherty *et al.*^38^ Individuals received three taste stimuli during fMRI imaging: 1 mol l^−1^ sucrose solution (100 trials), no solution (100 trials) and artificial saliva (80 trials). Individuals learned to associate each unconditioned taste stimulus (US) with a paired conditioned visual stimulus (CS) that is probabilistically associated with its US: the CS shape for sucrose was followed in 80% of trials by sucrose solution (the other 20% were followed by no solution), and the CS shape associated with no-solution (null) was followed in 80% of the trials by no solution (the other 20% were followed by sucrose); the CS shape for artificial saliva was always followed by saliva receipt. For each subject, the first 10 trials were fixed CS shape for sucrose followed by the delivery of US sucrose to establish an initial stable association between the CS sucrose shape and US sucrose taste.^38^ All other trials were fully randomized without predetermined order. The taste stimuli were applied using a customized-programmable syringe pump (J-Kem Scientific, St Louis, MO, USA) controlled with the E-Prime Software (Psychological Software Tools, Pittsburgh, PA, USA). Individual taste application was triggered by magnetic resonance imaging scanner radiofrequency pulse.^18^ Task duration was 28 min.

### Diffusion image analysis

Diffusion weighted images were processed using FSL's Diffusion Toolbox 4.1.3 (FDT, Oxford Centre for Functional MRI of the Brain, http://www.fmrib.ox.ac.uk/fsl). Images were corrected for eddy current distortions and head motion. Probabilistic fiber tractography was computed for each subject using PROBTRACKX2 to generate the most probable connectivity distribution between seed and ipsilateral target. Tractography parameters were as follows: 5000 sample tracts per seed voxel, 0.2 curvature threshold, step length of 0.5 and a maximum number of steps 2000. Connectivity was assessed by computing connection strength that determines the mean probability of streamlines for each seed–target combination. The calculated connection strength value was divided by the total connection probability of seed regions and then multiplied by the mean connection probability across seed and target regions and finally divided by the target volume of interest in order to normalize and rescale the results for size of seed and target regions.^39^ Physical path length was also corrected for.^39^ In each hemisphere, tract-based connection strength was calculated for anatomical white matter tracts connecting regions of a comprehensive taste reward hierarchy proposed by Rolls *et al.*^40^ (Supplementary Figure 1). Seed regions included the thalamus, dorsal anterior insula, ventral anterior insula, posterior insula, substantia nigra, central nucleus of the amygdala, basolateral amygdala, medial OFC, middle OFC, gyrus rectus and inferior OFC. Thalamus targets included all subregions of the insula and the frontal operculum. Targets of insula subregions included the basolateral amygdala, central nucleus of the amygdala, ventral striatum, medial prefrontal cortex (PFC), medial OFC, middle OFC, gyrus rectus and the inferior OFC. The ventral striatum was the target of the substantia nigra seed region. For both the central and the basolateral nucleus of the amygdala, the targets were the hypothalamus, substantia nigra, ventral striatum and the anterior cingulate cortex. OFC subregion targets included the hypothalamus, ventral striatum and the medial PFC. In total, tract-based connection strength was calculated for 98 white matter tracts connecting aforementioned seed and ipsilateral targets. The Automated Anatomical Labeling atlas was used to determine coordinates for each seed and target region.^41^

### fMRI analysis

Brain-imaging data were preprocessed and analyzed using the SPM8 software in Matlab R2009b, 7.9.0 (MathWorks, Natick, MA, USA). Data from each subject were realigned to the first volume, normalized to the Montreal Neurological Institute template and smoothed with a 6-mm full width at half maximum Gaussian kernel. Each image sequence was manually inspected, and images with artifacts or movement more than one voxel size were removed.

Data were modeled with a hemodynamic response function—convolved boxcar function—using the general linear model, including temporal and dispersion derivatives, and autoregression. A 128-s high-pass filter was applied to remove low-frequency fluctuation in the BOLD signal. Motion parameters were applied as regressors in the first-level analysis to correct for individual movement. We then developed first-level models in which we predicted the response in each voxel as a function of each of the stimulus conditions. For this study we computed the contrast for expected sucrose receipt versus expected receipt of no solution.

### Effective connectivity

Within SPM8, we extracted functional time-series data for expected receipt of 1 m sucrose solution for each of the seed and target regions of interest using the SPM marsbar toolbox. Effective connectivity was inferred using Independent Multiplesample Greedy Equivalence Search (IMaGES) and Linear non-gaussian Orientation, Fixed Structure search algorithms housed within the TETRAD V program.^42^ The goal of effective connectivity analyses is to understand causal relations among the neuronal populations whose activity gives rise to observed fMRI signals in spatially localized regions of interest. The results from those analyses are presented as directed graphs, where nodes or vertices in the graph represent brain regions and directed edges in the graph represent relatively direct causal influences of one region on another. The Independent Multiplesample Greedy Equivalence Search (IMaGES) is a modification of the Greedy Equivalence Search (GES) that allows to analyze multiple data sets. GES begins with an empty graph whose vertices are the recorded variables and proceeds to search forward, one new connection at a time, over Markov Equivalence classes of directed acyclic graphs. Each class of models with an additional edge is scored using the Bayes Information Criterion: −2ln(ML) + *k* ln (*n*), where ML is the maximum likelihood estimate, *k* is the dimension of the model (the number of directed edges plus the number of variables) and *n* is the sample size. The algorithm searches forward from the empty graph until no improvement in the Bayes Information Criterion score is possible, and then backward, and outputs a description of a Markov Equivalence class. In practice, the algorithm requires a computation of a series of maximum likelihood estimates, and is limited to cases where approximations to such estimates can be rapidly obtained. The analysis process in IMaGES and GES is nonlinear, and therefore a comparison of a parameterized output of the GES using conventional linear models for group comparison is not recommended. IMaGES was supplemented by a Linear non-gaussian Orientation, Fixed Structure algorithm postprocessor; this leads to a precision of orientations that is greater than 90% and the precision of recall greater than 80%, that is, more edges are directed than with IMaGES alone, and with no loss of accuracy.^25^

### Statistical analyses

Demographic and behavioral data were analyzed using SPSS 23.0 (IBM-SPSS, Chicago, IL, USA) using multivariate multivariate analyses of variance (MANOVAs); extracted data for brain connectivity were in addition corrected for medication use, anxiety and mood disorder diagnoses (added factors in the model) and *post hoc* pairwise between group analyses were Bonferroni-corrected. In addition, we analyzed the connectivity data with MANOVA without added factors as well as a non-parametric Kruskal–Wallis test because not all data in each group were normally distributed (Supplementary Table 2). Linear regression analyses to test behavior–brain relationships were applied for age, body mass index and 1 m sucrose ratings for pleasantness and sweetness; additional exploratory analyses tested the potentially confounding effects of depression or anxiety measures. Significant correlations were corrected using the false discovery rate using the method proposed by Benjamini and Hochberg.^43^

## Results

### Demographics and assessment results

Groups were matched for age; anorexia nervosa subjects had significantly (Table 1) lower body mass index compared with the other study groups; harm avoidance, depression, drive for thinness, body dissatisfaction, state and trait anxiety and sensitivity to punishment were higher in both eating-disorder groups compared with controls. Sensitivity to reward was greater compared with controls only in the bulimia nervosa group, whereas novelty-seeking was lower in the anorexia nervosa group.

### White matter connectivity strength

**Figure 1:**
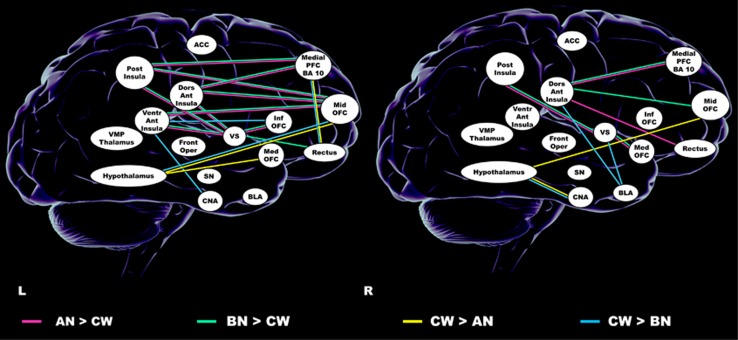
Connection strength results. ACC, anterior cingulate cortex; AN, anorexia nervosa; BLA, basolateral amygdala; BN, bulimia nervosa; CNA, central nucleus of the amygdala; CW, Controls; Dors Ant Insula, dorsal anterior insula; Front Oper, frontal operculum; Inf OFC, inferior orbitofrontal cortex; L, left; Med OFC, medial orbitofrontal Cortex; Medial PFC, BA 10, medial prefrontal cortex, Brodmann Area 10; Mid OFC, middle orbitofrontal cortex; Post Insula, posterior insula; R, right; Rectus, gyrus rectus; SN, substantia nigra; Ventr Ant Insula, ventral anterior insula; VS, ventral striatum; VMP Thalamus, ventral posterior medial thalamus.

Left: white matter connection strength (Figure 1 and Supplementary Table 1) was *higher* in both eating disorder groups between insula regions and middle OFC, between ventral and dorsal anterior insula to ventral striatum and dorsal anterior insula, from posterior insula to medial PFC, and between inferior OFC and gyrus rectus and ventral striatum. In anorexia and bulimia nervosa groups, connectivity strength was *lower* between middle OFC and hypothalamus, as well as between gyrus rectus and medial PFC. In bulimia nervosa only, connectivity was lower compared with controls from ventral anterior insula to inferior OFC and central nucleus of the amygdala, and in anorexia nervosa between medial OFC and hypothalamus.

Right: Connectivity strength was *greater* in both eating-disorder groups in tracts from posterior insula and medial OFC to ventral striatum and from dorsal anterior insula to the medial PFC.

Anorexia and bulimia nervosa groups showed *less* connectivity between basolateral nucleus of the amygdala and hypothalamus; anorexia nervosa had less connectivity between middle OFC and hypothalamus, and bulimia nervosa from the amygdala basolateral nucleus to the ventral striatum and dorsal anterior insula.

The additional analysis using MANOVA without comorbidity or medication as factors, or the analysis using the non-parametric test indicated that the MANOVA with added factors did not inflate results (Supplementary Table 1).

### Effective dynamic connectivity

**Figure 2:**
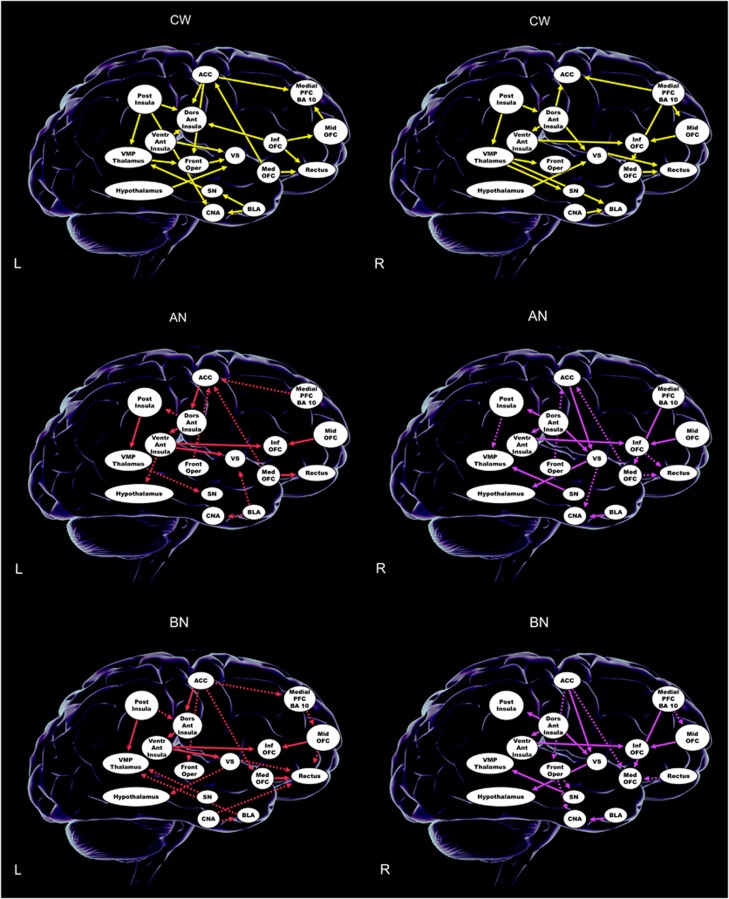
Effective connectivity. ACC, anterior cingulate cortex; AN, anorexia nervosa; BLA, basolateral amygdala; BN, bulimia nervosa; CNA, central nucleus of the amygdala; CW, Controls; Dors Ant Insula, dorsal anterior insula; Front Oper, frontal operculum; Inf OFC, inferior orbitofrontal cortex; L, left; Med OFC, medial orbitofrontal cortex; Medial PFC, BA 10, medial prefrontal cortex, Brodmann Area 10; Mid OFC, middle orbitofrontal cortex; Post Insula, posterior insula; R, right; Rectus, gyrus rectus; SN, substantia nigra; Ventr Ant Insula, ventral anterior insula; VMP Thalamus, ventral posterior medial thalamus; VS, ventral striatum. For AN and BN, solid lines indicate similar pattern and dashed lines indicate different pattern between AN and BN groups. Yellow lines are used for left- and right-sided connections in the CW. For the AN and BN groups, red lines indicate left-sided connections and purple lines indicate right-sided connections.

There were bilateral effective connectivity patterns that all groups (Figure 2 and Table 2) shared: on the left from insula regions to the thalamus and ventral striatum, and on the right from the ventral anterior insula to OFC as well as intra-insular and orbitofrontal connections.

Only the controls had an effective connectivity pattern from the hypothalamus to ventral striatum bilaterally. On the right side both eating-disorder groups showed effective connectivity from the anterior cingulate to ventral striatum, and from there to the hypothalamus. Substantia nigra effectively connected to the thalamus, whereas the opposite relationship was the case in controls.

Unique to anorexia nervosa was left- and right-sided connectivity driven by the frontal operculum to the anterior cingulate cortex; the bulimia nervosa group showed a unique pattern of dynamic connectivity from anterior cingulate to medial OFC. Both anorexia and bulimia nervosa showed effective connectivity on the left from ventral anterior insula to inferior OFC, middle to inferior OFC and dorsal to ventral anterior insula.

### Correlation analyses

Age, body mass index or pleasantness perception were not significantly correlated with brain results in any group or pathway after false discovery rate correction. All groups showed positive correlations between sweetness perception and connection strength in fibers that terminated in the middle OFC, although right hemispheric in controls and left-sided in eating-disorder groups (Table 3). In addition, in the anorexia nervosa group only, there was a pattern of negative correlation for pathways between thalamus, hypothalamus, insula and limbic brain regions. Duration of illness was positively correlated with connection strength for five pathways in the eating-disorder groups, but none survived false discovery rate correction.

To test for confounding variables, we tested whether group differences in regional brain response to sucrose solution was related to connection strength or effective connectivity. We conducted an additional whole-brain contrast for expected sucrose against no solution receipt. This analysis did not show any group differences (*P<*0.001, 10 voxel cluster threshold). In addition, we calculated FA for investigated fiber paths using fslstats,^30^ and tested whether FA predicted group differences for connectivity strength or effective connectivity. FA was lower (*P<*0.05, corrected for multiple comparisons, comorbidity and medication) in both anorexia and bulimia groups compared with controls from left ventral anterior insula and gyrus rectus to ventral striatum, left posterior insula to middle OFC and right middle OFC to hypothalamus. In anorexia nervosa only, FA was lower in connections from right central nucleus of amygdala to hypothalamus, left dorsal anterior insula to ventral striatum, right dorsal anterior insula to gyrus rectus, bilateral posterior insula to ventral striatum, left medial OFC to hypothalamus, right medial OFC to ventral striatum and left gyrus rectus to PFC. There were no significant correlations between FA and structural connectivity, and effective connectivity was not selectively altered in pathways with lower FA.

## Discussion

The results of this study indicate that both anorexia and bulimia nervosa are associated with widespread alterations in white matter structural as well as effective connectivity in taste-reward and appetite-regulating pathways. Structural connectivity was greater in both eating-disorder groups between insula and orbito- and PFC regions, whereas connection strength was lower in pathways to the hypothalamus, a region central to feeding regulation. Effective connectivity during sweet taste stimulation differed between groups, including the anterior cingulate showing effective connectivity to the ventral striatum, which modulated hypothalamus activity in anorexia and bulimia nervosa, whereas in controls the hypothalamus was driving ventral striatal activity. White matter structural connectivity was positively correlated with sweet taste perception in all groups in pathways that terminated in the middle OFC, but anorexia nervosa showed an additional negative correlation with connectivity strength between thalamus, hypothalamus and insula.

Human brain imaging research in anorexia and bulimia nervosa has made progress over the past years and has repeatedly implicated brain taste-reward and salience-processing regions in the pathophysiology of those disorders. Whether there are circuitry differences in structural white matter fiber connectivity and thus organization, or whether the effective functional interactions of those brain regions differ in eating disorders compared with controls had not been studied. Here we wanted to test whether greater structural white matter connectivity is a common marker of white matter organization across taste- and appetite-regulating pathways in eating disorders. Our results now indicate that anorexia and bulimia nervosa during the ill state are associated with bilaterally higher connectivity between insula, frontal cortex and ventral striatum, but on the contrary with lower connectivity between OFC and hypothalamus on the left, and lower connectivity between basolateral nucleus of the amygdala and hypothalamus on the right; anorexia nervosa showed lower connectivity between OFC and hypothalamus pathways bilaterally. Those results suggest that taste-reward circuitry alterations in eating disorder go beyond connections between insula, OFC and ventral striatum and include the hypothalamus, a key structure in appetite control.^44^ Animal studies have shown that the connections from the cortex and amygdala to the hypothalamus are important for cue-mediated food intake, or how much an individual eats after presentation of feeding-associated stimuli.^45^ Research on learning, operant conditioning and food avoidance in eating disorders is sparse. Research has shown that humans are ‘innately' programmed to like sweet tastes at birth.^46, 47^ Individuals with eating disorders typically start to avoid, for instance, eating sweets because they are afraid of gaining weight. One could see such avoidance as a form of learned behavior, and more specifically operant conditioning, with weight gain as the feared ‘punishment'.^48^ Thus, altered functioning in frontohypothalamic circuits could facilitate or inhibit operant conditioning or reversal of such associations. Psychotherapy for meal support and nutritional rehabilitation is designed to re-establish normal eating patterns and tolerate the feared stimulus, food. However, whether those processes indeed follow the principles of operant conditioning has been insufficiently studied and deserves further exploration. The reward-system crosstalk between hypothalamus, striatum and amygdala involves neurotransmitters such as dopamine, gamma-amino-butyric acid, glutamate and orexin,^49^ and animal models and neurotransmitter receptor studies will be needed to further understand neurochemical alterations that could alter neural transmission.

The combination of greater connectivity and lower FA is striking. We have found a similar phenomenon in women recovered from anorexia nervosa previously.^30^ FA is the scalar composite of axial and radial diffusivity, giving information on water diffusion across the various directions along paths. The connectivity on the other hand is a probability measure for how many fibers may connect a seed with a target region, without emphasis on radial diffusivity. Thus, the connectivity is a reflection of the number of connections that go from one point to another, whereas the FA value is thought to represent the structural integrity of those fibers. Previously, higher connectivity has been described in, for instance, in dementia of the Alzheimer's type, a condition that is by the same time associated with lower FA.^50^ It is possible that altered white matter connectivity is compensatory to effects from the illness on white matter integrity. In our previous study in anorexia nervosa after recovery, we found that longer duration of illness predicted greater connectivity strength.^30^ In this study we found some indication for a similar relationship, but less strongly not surviving multiple comparison correction. This could be due to the fact that subjects were still in the ill phase, and with ongoing illness this relationship may strengthen. We are currently recruiting subjects from this cohort when recovered in order to shed further light on this question.

To the best of our knowledge, this is the first study that provides evidence that structural white matter connectivity is related to individual sweet-taste perception. In all three groups, connectivity between middle OFC and insula was positively correlated with subjective sweetness perception, although this was found on the right in controls and in the eating-disorder groups on the left. The mechanistic underpinnings for such a relationship are elusive and require further study. In addition, in anorexia nervosa, structural connectivity correlated negatively with sweet perception between the ventromedial posterior nucleus of the thalamus and the insula, as well as fiber paths originating from the left amygdala and connections between left posterior insula and gyrus rectus. The mean connection strength between anorexia nervosa and controls between those regions was not different, and the implications of this negative correlation are speculative.

The effective conectivity, or dynamic causal mechanisms of brain circuits' interaction during tasting sucrose solution showed similarities but also fundamentally different patterns between groups. All groups showed bilaterally effective connectivity from insula regions to ventral striatum and OFC, as well as prefrontal—OFC connectivity on the right side. We are not aware that this has been studied previously in this manner in humans. Taste information is thought to be transmitted via the thalamus to insula/frontal operculum and from there to anterior cingulate, ventral striatum, OFC and hypothalamus;^51^ however, new research using salient stimuli suggests a greater complexity of those pathways.^52^ One study suggested effective connectivity based on attention to monosodium glutamate taste intensity (PFC to insula) versus pleasantness (PFC to OFC).^53^ Our data now provide new information how sweet taste may activate the taste-reward system. In controls, bilaterally, the flux of activation was directed from the hypothalamus to ventral striatum, suggesting that hypothalamic signals have an important input on ventral striatal activation and maybe motivation to approach food stimuli. In contrast, in both anorexia and bulimia nervosa the right anterior cingulate-effective connectivity was directed to the ventral striatum, which in turn mediated hypothalamus activity. This reversal of input may have key effects on appetite regulation in eating disorders. Basic science suggests that hypothalamus–ventral striatum connections are important for feeding regulation.^54^ Our data suggest that in eating disorders there may be a top–down control of this circuitry. The anterior cingulate is important in error-monitoring and anxiety-processing,^55^ and eating-related fearful cognitions could have an impact on subcortical taste-reward processing, which in turn could alter the normal hypothalamic feeding-drive input.

In anorexia nervosa, bilaterally effective connectivity was directed from frontal operculum to the anterior cingulate, and bulimia nervosa showed anterior cingulate-effective connectivity to the medial OFC. Input from the frontal operculum to the anterior cingulate could reinforce food avoidance behavior and anterior cingulate–OFC-effective connectivity could have an impact on value computation of sweet taste perception or hedonic experience in bulimia nervosa; however, testing this hypothesis will require a specific study design.^53^

We did not see overlap between areas of altered group-effective connectivity and structural connectivity strength. This suggests that those results and potential function–behavior implications are independent. On the other hand, altered white matter connectivity strength could be compensatory and normalize effective dynamic connectivity. White matter integrity was lower in most pathways tested in the anorexia but only in few white matter tracts in bulimia nervosa compared with controls. One interpretation is that FA could be related to the severity of malnutrition. This will need further study and across time from illness through the recovery process.

### Limitations

The sample size was modest and requires replication. However, partial eta squared for connectivity strength between group comparisons was >0.5, indicating large effect size. Probabilistic tractography does not provide absolute fiber counts.^24^ Still, this method does provide results comparable to direct white matter neuron-tracing,^56^ suggesting that our results are valid. Utilizing 25 diffusion directions during MRI data acquisition may limit probabilistic tractography analysis; however, it has been shown that increasing diffusion directions does not improve fiber-tracking.^57^ The mechanism of greater white matter connectivity in eating disorders is uncertain, but could be due to white matter reorganization after tissue injury.^50^ Future studies will need to further explore whether alterations found result from underweight or are premorbid traits. The IMaGES algorithm used here may be one of the most reliable tools available,^42^ but it cannot describe whether the effective connectivity is increasing or decreasing activity between connected regions. The effective connectivity analysis found across-group connectivity in the same or opposite direction, or effective connectivity between regions in one group but not in another, and a limitation of the method is that direct comparison of connectivity parameters across groups cannot reliably be done based on those connectivity patterns. A limitation of this study is the cross-sectional design, and we are currently studying individuals with anorexia nervosa during recovery and hope that this will help better describe potential underlying mechanisms. This is a typical clinical eating-disorder sample with typical comorbidity of anxiety and depressive disorders. In order to control for those effects we included comorbidity (anxiety disorder and major depressive disorder) and medication use (selective serotonin reuptake inhibitor and antipsychotic) in the statistical model as factors but we cannot entirely rule out the impact of the comorbid conditions on the results. Anxiety and depression ratings did not significantly correlate with connectivity measures. In order to understand the mechanisms of connectivity strength as well as effective connectivity, it will be important to study those variables at different time points during disease and recovery. Ideally, a longitudinal approach is chosen. One very recent study exists that investigated effective connectivity and connectivity strength before and after weight restoration. That study included only one fiber path, the connection from the nucleus accumbens to the OFC, which was increased at both time points.^29^ Effective connectivity is an up to date less commonly used technique. We still are just at the beginning of understanding how brain regions interact, and we cannot exclude that effects from comorbid conditions had an impact on the effective connectivity results. In depression, for instance, a study found greater connectivity within the anterior cingulate cortex during a cognitive task compared with controls.^58^ Another study reported on lower prefrontal cortical–amygdala-effective connectivity in response to negative emotional faces in women with postpartum depression. In addition, a study in youth indicated that adolescents with major depressive disorder had lower effective connectivity from the amygdala to the anterior cingulate.^59^ In a study in social anxiety disorder the individuals with anxiety showed connectivity from the OFC to the amygdala, which was not observed in controls.^60^ Because those studies did not use taste stimuli or focused on, for instance, hypothalamus circuitry they are not comparable with this study. However, a study in controls showed that taste stimulation was related to effective connectivity from the insular cortex to the thalamus, a direction that we observed in our study subjects as well.^61^

## Conclusion

This study suggests greater white matter connection strength across frontostriatal reward pathways, but reduced connectivity strength to the hypothalamus, which could have important implications on appetite regulation. The effective network connectivity from anterior cingulate to ventral striatum and to the hypothalamus in eating disorders provides a possible biological correlate for the hypothesis that those individuals are able to override homeostatic signals.

## Figures and Tables

**Table 1:** Demographic and behavioral data

	*CW (*n=*26)*		*AN (*n=*26)*		*BN (*n=*25)*		*MANOVA analysis*		
	*Mean*	*s.d.*	*Mean*	*s.d.*	*Mean*	*s.d.*	*F*	P	*Comparison*
Age (years)	24.39	3.49	23.23	5.26	24.64	4.22	0.75	0.474	N.S.
Body mass index (kg/m^2^)	21.61	1.21	16.23	1.09	23.56	5.89	30.42	<0.001	CW>AN*** BN>AN***
Novelty-seeking	18.42	5.27	13.89	6.02	22.20	6.70	12.24	<0.001	CW>AN* BN>AN***
Harm avoidance	10.08	4.74	23.65	5.94	22.48	5.93	47.36	<0.001	AN>CW*** BN>CW***
Reward dependence	16.54	3.47	15.04	3.04	15.60	4.57	1.07	0.349	N.S.
Depression (BDI)	1.27	1.28	21.27	12.94	22.68	14.58	29.34	<0.001	AN>CW*** BN>CW***
Drive for thinness (EDI-3)	2.42	3.51	19.96	5.98	21.92	4.65	127.73	<0.001	AN>CW*** BN>CW***
Bulimia (EDI-3)	0.92	1.23	3.89	4.97	20.00	5.43	144.84	<0.001	AN>CW* BN>CW*** BN>AN***
Body dissatisfaction (EDI-3)	4.62	4.26	24.31	8.99	30.44	7.58	89.78	<0.001	AN>CW*** BN>CW*** AN>BN*
Sensitivity to reward	5.00	2.95	6.69	3.71	7.56	3.38	3.84	0.026	BN>CW*
Sensitivity to punishment	4.42	2.69	12.96	3.85	12.56	3.80	49.49	<0.001	AN>CW*** BN>CW***
State anxiety	26.46	4.82	55.23	11.95	50.52	13.14	55.04	<0.001	AN>CW*** BN>CW***
Trait anxiety	28.04	4.29	56.39	11.98	58.80	9.75	88.17	<0.001	AN>CW*** BN>CW***
1 M Sucrose pleasantness	4.92	2.43	4.58	2.32	5.88	2.57	1.94	0.151	N.S.
1 M Sucrose sweetness	8.23	0.82	8.31	1.19	8.28	0.98	0.04	0.962	N.S.
Education (years)	16.52	1.92	14.39	2.25	15.77	3.09	4.65	0.013	CW>AN**
Duration of illness (years)	—	—	6.62	5.65	7.08	4.51	—	—	—
									
	N		N		N				
Oral contraceptive use	16		6		2				
Antidepressant use	0		13		16				
Antipsychotic use	0		3		5				
Major depression	0		4		4				
Anxiety disorder	0		5		6				
Major depression and anxiety disorder	0		10		13				

Abbreviations: AN, anorexia nervosa; BDI, Beck Depression Inventory; BN, bulimia nervosa; CW, control women; EDI-3, Eating Disorder Inventory-3; MANOVA, multivariate analysis of variance; N.S., non significant.

*P<0.05, **P<0.01, ***P<0.001. Significance is based on the Dunnett's T3 *post hoc* test.

**Table 2:**
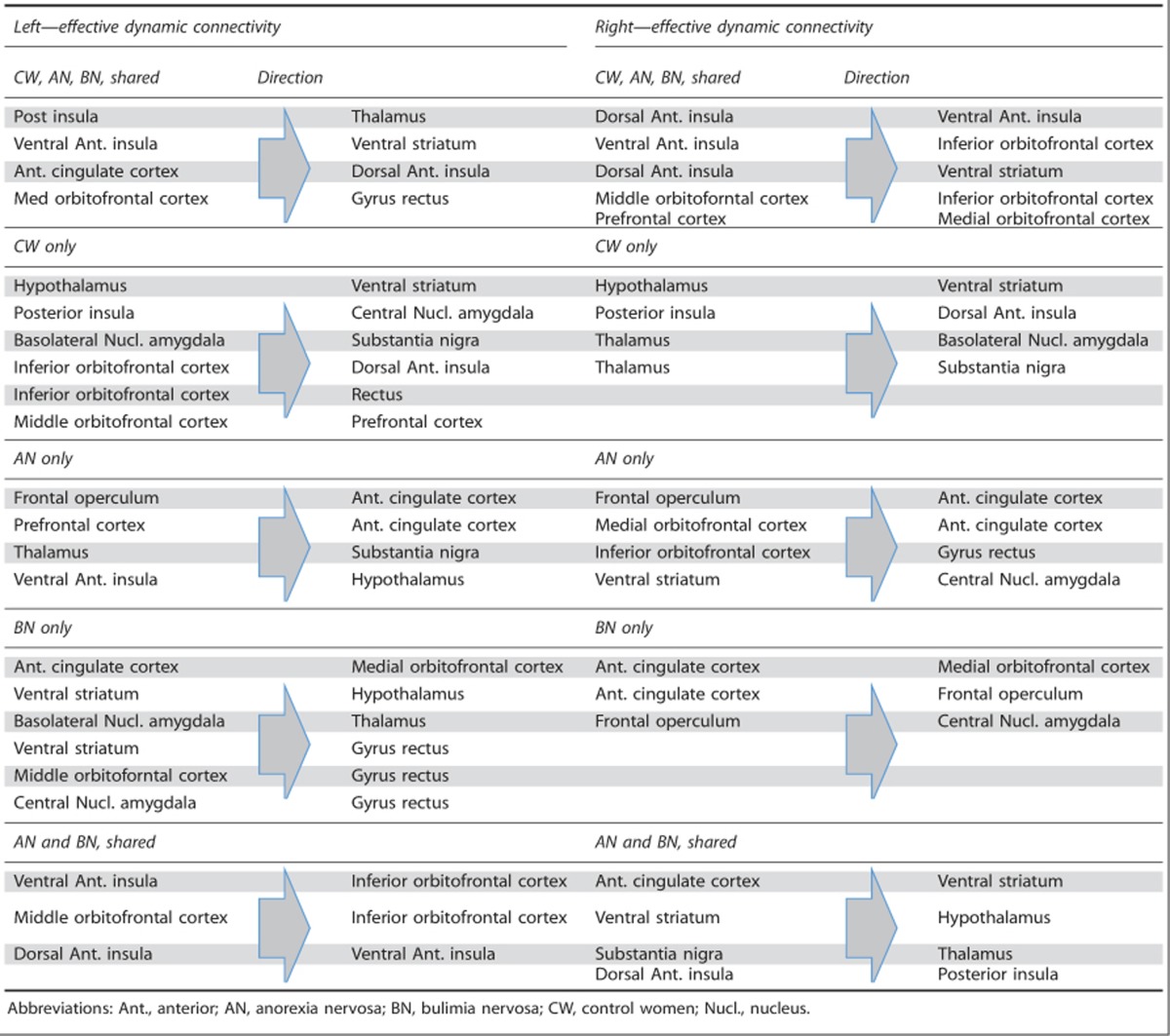
Effective connectivity results

**Table 3:** Correlation between connection strength and 1 m sucrose sweetness ratings

*Correlation sweetness rating—connection strength*			r	P
CW	R posterior insula	Middle OFC	0.531	0.021
	R ventral anterior insula	Middle OFC	0.501	0.037
AN	L dorsal anterior insula	Middle OFC	0.586	0.038
	L inferior OFC	Prefrontal cortex	0.737	<0.001
	L central nucleus amygdala	ACC	−0.766	<0.001
	L central nucleus amygdala	Hypothalamus	−0.774	<0.001
	L posterior insula	OFC gyrus rectus	−0.605	0.024
	L medial OFC	Hypothalamus	−0.732	<0.001
	R medial OFC	Hypothalamus	−0.741	<0.001
	L middle OFC	Hypothalamus	−0.601	0.027
	L substantia nigra	Ventral striatum	−0.764	<0.001
	L ventral medial posterior nucleus	Ventral anterior insula	−0.637	0.011
	R ventral medial posterior nucleus	Dorsal anterior insula	−0.650	0.007
BN	L dorsal anterior insula	Middle OFC	0.534	0.042
	L ventral anterior insula	Middle OFC	0.572	0.020

Abbreviations: ACC, anterior cingulate cortex; AN, anorexia nervosa; BN, bulimia nervosa; CW, control women; FDR, false discovery rate; L, left; OFC, orbitofrontal cortex; R, right.

All presented *P* values are after FDR correction.
