# Supplementary material for: Altered structural and effective connectivity in anorexia and bulimia nervosa in circuits that regulate energy and reward homeostasis
Source: Transl Psychiatry. 2016 Nov 1;6(11):e932–. doi: 10.1038/tp.2016.199 (PMC5314116; doi:10.1038/tp.2016.199)
Supplement: Supplementary Material [file tp2016199x1.pdf]

Supplemental Material

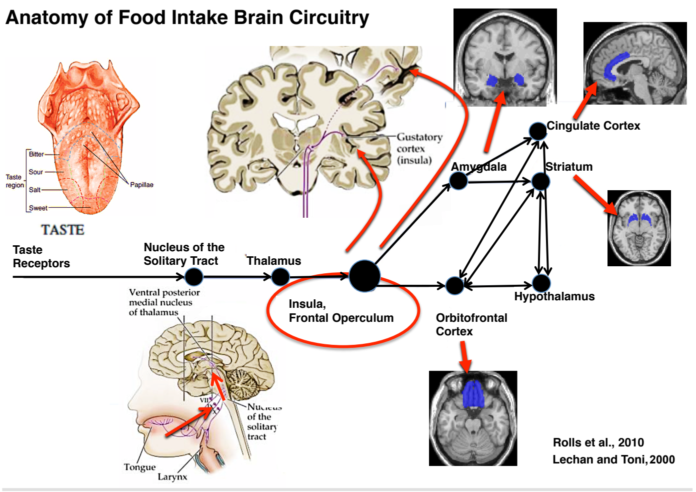

Supplemental Figure 1. Anatomical connections between taste and food reward circuitry based on Rolls et al. 2010 and Lechan and Toni 2000.

Rolls ET. The affective and cognitive processing of touch, oral texture, and temperature in the brain. Neurosci Biobehav Rev. 2010;34(2):237-45. Epub 2008/05/13. doi: S0149-7634(08)00046-8 [pii] 10.1016/j.neubiorev.2008.03.010. PubMed PMID: 18468687.

Lechan RM, Toni R. Functional Anatomy of the Hypothalamus and Pituitary. In: De Groot LJ, Beck-Peccoz P, Chrousos G, Dungan K, Grossman A, Hershman JM, et al., editors. Endotext. South Dartmouth (MA)2000.

Movement Parameters:

All subjects' movements of the head during fMRI were within the 1-voxel threshold and no subject was removed. Movement parameters were included in the preprocessing analysis. There were no statistical differences between movement parameters between groups (Supplemental Table 1) and no subject exceeded movement of one voxel (3mm).

	HC		AN		BN			
**Movement**	**M**	**SD**	**M**	**SD**	**M**	**SD**	**F**	**p**
X translation	-0.17776	0.67373	-0.03708	0.43703	0.08163	0.63373	1.185	0.312
Y translation	0.31773	0.39516	0.49457	0.31240	0.47708	0.29853	2.086	0.132
Z translation	0.10157	1.12886	-0.43239	1.15919	-0.20124	1.17117	1.378	0.259
Roll rotation	0.00374	0.02032	0.01046	0.02221	0.00381	0.01762	0.905	0.409
Pitch rotation	-0.00397	0.01644	0.00109	0.01226	-0.00196	0.01176	0.875	0.421
Yaw rotation	-0.00002	0.00641	0.00030	0.00613	0.00091	0.00867	0.108	0.898

Supplemental Table 1. Movement parameters across groups.

fMRI Main Effects and Comparison Between Groups

fMRI contrast main effect data for the contrast Receiving Sucrose vs. Receiving No Solution per ROI were also extracted and compared across groups. The data were non-normally distributed, therefore rank transformed and analyzed by group including the same cofactors as in the analysis of connectivity strength. The multivariate Wilks' Lambda for group effect was 0.390, $p<0.986$; individual group effects by region were non significant.

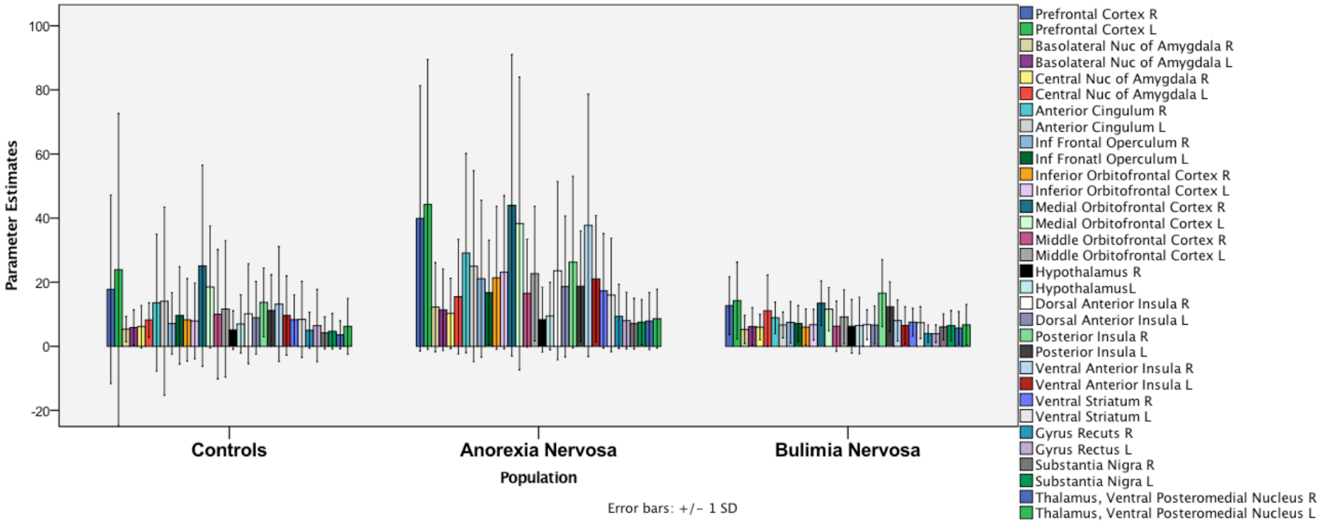

Supplemental Figure 2. Graphical representation of main effect parameter estimates by group and region of interest for the contrast receiving sucrose vs. receiving no solution.

							With Co Factors			No Cofactors			Nonparametric Test		
	CW (n=26)		AN (n=26)		BN (n= 25)		MANOVA			MANOVA			Kruskal-Wallis Test		
	Mean	SD	Mean	SD	Mean	SD	F	p		F	p		Test Statistic	otic Sig (2-sided test)	
BLA - ACC, L	0.00	0.00	9.69	49.39	0.00	0.00	1.787	0.175		0.980	0.380		1.962	0.375	
BLA - Hypothalamus, L	18.44	86.62	2.17	9.94	0.00	0.00	0.548	0.580		1.019	0.366		8.135	0.017	CW>BN*
BLA - SN, L	13.56	62.13	0.72	2.28	0.66	3.29	0.434	0.650		1.089	0.342		3.938	0.140	
BLA - Ventral Striatum, L	0.39	1.48	27.90	142.27	0.00	0.00	1.798	0.173		0.966	0.385		9.021	0.011	CW>BN*
BLA - ACC, R	0.01	0.02	0.01	0.03	0.00	0.01	3.516	0.035		1.430	0.246		2.828	0.243	
BLA - Hypothalamus, R	0.42	1.57	1.82	5.16	0.15	0.73	2.427	0.096		2.066	0.134		1.180	0.554	
BLA - SN, R	0.40	1.14	0.94	2.89	0.28	0.97	1.839	0.166		0.891	0.414		0.273	0.872	
BLA - Ventral Striatum, R	0.38	0.50	0.56	1.41	0.05	0.14	4.880	0.010	CW>BN*	2.221	0.116		10.075	0.006	CW>BN**
CNA- ACC, L	0.09	0.24	0.04	0.12	0.01	0.03	0.773	0.465		1.820	0.169		4.428	0.109	
CNA - Hypothalamus, L	254.84	1238.30	4.64	14.33	3.38	16.07	0.388	0.680		1.045	0.357		14.058	0.001	CW>BN***
CNA - SN, L	320.62	724.54	115.72	468.01	45.97	183.56	1.873	0.161		1.995	0.143		18.701	<0.001	CW>BN***; AN>BN*
CNA - Ventral Striatum, L	28.78	116.12	3.96	12.66	0.02	0.09	0.620	0.541		1.358	0.263		28.430	<0.001	CW>AN**; CW>BN***
CNA - ACC, R	0.07	0.26	0.02	0.07	0.02	0.09	0.799	0.454		0.746	0.478		4.081	0.130	
CNA - Hypothalamus, R	3.28	3.68	1.43	3.33	0.30	1.03	5.505	0.006	CW>AN*; CW>BN**	6.697	0.002	CW>BN**	16.158	<0.001	CW>AN*; CW>BN***
CNA - SN, R	1.92	3.52	2.60	5.48	0.71	2.91	2.090	0.131		1.364	0.262		4.139	0.126	
CNA - Ventral Striatum, R	1.28	1.44	2.77	9.52	0.16	0.35	3.614	0.032		1.403	0.252		16.284	<0.001	CW>AN*; CW>BN***
Inferior OFC - BA10, L	13.84	2.49	14.95	1.22	15.18	0.06	1.357	0.264		5.064	0.009	AN>CW*; BN>CW*	23.293	<0.001	AN>CW***; BN>CW***
Inferior OFC - Hypothalamus, L	70.29	137.57	66.70	156.65	40.84	72.12	0.187	0.830		0.399	0.672		3.085	0.214	
Inferior OFC - Ventral Striatum, L	81.06	32.01	104.04	27.50	111.90	16.50	3.972	0.023	BN>CW*	9.560	<0.001	AN>CW**; BN>CW***	18.815	<0.001	AN>CW*; BN>CW***
Inferior OFC - BA10, R	11.00	3.74	12.87	3.68	13.41	3.13	0.863	0.426		3.292	0.043	no post hoc difference	6.637	0.036	BN>CW*
Inferior OFC - Hypothalamus, R	26.63	46.84	74.37	178.42	35.90	94.48	1.717	0.187		1.154	0.321		0.425	0.809	
Inferior OFC - Ventral Striatum, R	58.96	41.67	89.66	38.45	96.14	44.57	2.266	0.111		5.865	0.004	AN>CW*; BN>CW**	10.602	0.005	BN>CW**
Dorsal Anterior Insula - BA10, L	12.47	10.41	27.03	6.30	24.71	8.77	7.653	0.001	AN>CW***; BN>CW*	21.140	<0.001	AN>CW***; BN>CW***	22.275	<0.001	AN>CW***; BN>CW***
Dorsal Anterior Insula - BLA, L	0.06	0.17	0.02	0.07	0.00	0.00	1.565	0.216		2.361	0.101		5.777	0.056	
Dorsal Anterior Insula - CNA, L	1.69	3.57	0.32	1.01	0.04	0.12	2.156	0.123		4.267	0.018	CW>BN*	24.974	<0.001	CW>AN***; CW>BN***
Dorsal Anterior Insula - Inferior OFC, L	32.26	0.00	32.26	0.00	30.96	6.45	1.152	0.322		1.045	0.357		4.304	0.116	
Dorsal Anterior Insula - Medial OFC, L	1.23	4.23	1.35	5.13	2.03	9.07	0.081	0.922		0.113	0.893		6.930	0.031	AN>BN*
Dorsal Anterior Insula - Middle OFC, L	15.46	18.14	42.12	20.25	37.04	20.14	9.982	<0.001	AN>CW***; BN>CW**	13.621	<0.001	AN>CW***; BN>CW***	16.878	<0.001	AN>CW***; BN>CW*
Dorsal Anterior Insula - Rectus, L	0.59	1.54	0.60	1.31	0.84	2.56	0.239	0.788		0.147	0.863		1.686	0.430	
Dorsal Anterior Insula - Ventral Striatum, L	122.73	77.23	195.63	56.54	199.77	55.61	5.026	0.009	AN>CW**; BN>CW*	11.823	<0.001	AN>CW***; BN>CW***	15.617	<0.001	AN>CW**; BN>CW***
Dorsal Anterior Insula - BA10, R	17.36	5.90	20.95	7.16	21.15	6.11	3.701	0.030	AN>CW*; BN>CW*	2.861	0.064		10.187	0.006	AN>CW*; BN>CW*
Dorsal Anterior Insula - BLA, R	0.14	0.24	0.03	0.07	0.01	0.02	3.541	0.034	CW>BN*	6.568	0.002	CW>AN*; CW>BN**	13.647	0.001	CW>BN***
Dorsal Anterior Insula - CNA, R	1.60	4.27	0.07	0.11	0.01	0.03	1.315	0.275		3.410	0.038		34.686	<0.001	CW>AN***; CW>BN***
Dorsal Anterior Insula - Inferior OFC, R	26.80	0.00	26.80	0.00	26.80	0.00	100% of connectivity probability between regions and across groups								
Dorsal Anterior Insula - Medial OFC, R	7.09	15.15	16.79	19.28	10.51	16.98	1.775	0.177		2.119	0.127		9.682	0.008	AN>CW**
Dorsal Anterior Insula - Middle OFC, R	30.01	15.82	37.01	17.49	41.57	13.69	3.042	0.054		3.487	0.036	BN>CW*	9.894	0.007	BN>CW**
Dorsal Anterior Insula - Rectus, R	2.81	8.44	10.53	14.34	6.73	10.16	4.199	0.019	AN>CW*	3.044	0.054		18.035	<0.001	AN>CW***; BN>CW***
Dorsal Anterior Insula - Ventral Striatum, R	152.36	55.20	158.56	57.83	160.17	60.72	0.021	0.979		0.130	0.878		0.656	0.721	
Posterior Insula - BA10, L	1.76	3.90	6.96	5.94	7.59	7.30	4.700	0.012	AN>CW*; BN>CW*	7.677	0.001	AN>CW**; BN>CW**	23.827	<0.001	AN>CW***; BN>CW***
Posterior Insula - BLA, L	0.10	0.34	0.01	0.02	0.00	0.01	0.878	0.420		2.170	0.121		2.997	0.223	
Posterior Insula - CNA, L	4.58	17.51	6.39	22.22	0.02	0.06	1.727	0.185		1.011	0.369		23.513	<0.001	CW>BN***; AN>BN*
Posterior Insula - Inferior OFC, L	5.47	6.53	10.24	7.49	9.06	7.71	1.120	0.332		3.048	0.053		6.943	0.031	AN>CW*
Posterior Insula - Medial OFC, L	0.10	0.21	0.47	1.27	0.11	0.45	1.617	0.206		1.806	0.171		8.664	0.013	CW>BN*; AN>BN*
Posterior Insula - Middle OFC, L	3.65	9.47	13.20	12.61	11.58	12.83	5.045	0.009	AN>CW**; BN>CW*	4.924	0.010	BN>CW*	24.632	<0.001	AN>CW***; BN>CW***
Posterior Insula - Rectus, L	0.07	0.10	0.95	2.71	0.07	0.12	2.323	0.105		2.691	0.074		7.601	0.022	AN>BN*
Posterior Insula - Ventral Striatum, L	57.61	61.99	142.66	46.59	130.56	44.42	13.418	<0.001	AN>CW***; BN>CW***	20.525	<0.001	AN>CW***; BN>CW***	24.583	<0.001	AN>CW***; BN>CW**
Posterior Insula - BA10, R	9.07	6.29	11.73	6.63	11.63	7.13	1.147	0.324		1.311	0.276		2.789	0.248	
Posterior Insula - BLA, R	0.39	1.19	0.08	0.21	0.02	0.06	0.580	0.562		2.050	0.136		9.846	0.007	CW>BN**
Posterior Insula - CNA, R	2.15	4.69	0.85	1.82	0.08	0.18	0.769	0.467		3.264	0.044	CW>BN**	9.161	0.010	CW>BN**
Posterior Insula - Inferior OFC, R	16.59	5.49	15.90	6.51	15.37	6.65	0.241	0.786		0.248	0.781		0.233	0.890	
Posterior Insula - Medial OFC, R	5.63	11.48	10.12	14.10	8.05	12.62	0.724	0.488		0.803	0.452		5.269	0.072	
Posterior Insula - Middle OFC, R	21.59	12.12	21.77	15.47	27.64	14.84	1.268	0.288		1.486	0.233		4.410	0.110	
Posterior Insula - Rectus, R	5.23	9.97	8.86	12.96	8.77	10.67	0.396	0.674		0.869	0.424		4.612	0.100	
Posterior Insula - Ventral Striatum, R	70.31	62.17	139.26	53.36	164.21	31.12	4.604	0.013	AN>CW*; BN>CW**	23.512	<0.001	AN>CW***; BN>CW***	29.665	<0.001	AN>CW***; BN>CW***
Ventral Anterior Insula - BA10, L	20.68	18.38	44.78	26.14	42.91	26.42	1.322	0.273		8.137	0.001	AN>CW**; BN>CW**	13.768	0.001	AN>CW**; BN>CW**
Ventral Anterior Insula - BLA, L	0.07	0.11	0.05	0.16	0.00	0.00	2.976	0.057		2.695	0.074		9.078	0.011	CW>BN**
Ventral Anterior Insula - CNA, L	0.96	1.50	0.41	1.00	0.05	0.14	3.500	0.036	CW>BN*	4.912	0.010	CW>BN**	16.444	<0.001	CW>BN***
Ventral Anterior Insula - Inferior OFC, L	0.05	0.08	0.02	0.05	0.00	0.01	3.500	0.036	CW>BN*	4.912	0.010	CW>BN**	16.444	<0.001	CW>BN***
Ventral Anterior Insula - Medial OFC, L	15.20	31.31	2.24	2.89	8.10	25.74	1.469	0.237		1.995	0.143		12.427	0.002	CW>AN*; CW>BN**
Ventral Anterior Insula - Middle OFC, L	56.48	40.17	116.23	32.83	103.38	38.27	14.425	<0.001	AN>CW***; BN>CW***	18.533	0.000	AN>CW***; BN>CW***	24.251	<0.001	AN>CW***; BN>CW***
Ventral Anterior Insula - Rectus, L	28.71	38.37	16.97	26.53	43.63	55.17	2.671	0.076		2.638	0.078		3.653	0.161	
Ventral Anterior Insula - Ventral Striatum, L	314.12	206.49	520.62	116.55	556.27	24.97	9.506	<0.001	AN>CW***; BN>CW***	22.923	<0.001	AN>CW***; BN>CW***	24.322	<0.001	AN>CW***; BN>CW***
Ventral Anterior Insula - BA10, R	48.71	19.72	41.32	26.13	31.26	26.92	2.369	0.101		3.269	0.044	CW>BN**	5.893	0.053	
Ventral Anterior Insula - BLA, R	0.40	0.73	0.07	0.17	0.01	0.05	3.121	0.050		6.052	0.004	CW>AN*; CW>BN**	14.178	0.001	CW>BN***; AN>BN*
Ventral Anterior Insula - CNA, R	3.30	8.17	0.30	1.07	0.02	0.07	1.766	0.179		3.711	0.029		23.781	<0.001	CW>AN**; CW>BN***
Ventral Anterior Insula - Inferior OFC, R	78.97	0.00	78.97	0.00	78.97	0.00	100% of connectivity probability between regions and across groups								
Ventral Anterior Insula - Medial OFC, R	37.33	53.04	48.78	60.10	42.10	65.32	0.272	0.763		0.242	0.786		1.037	0.596	
Ventral Anterior Insula - Middle OFC, R	117.45	35.65	112.36	45.03	116.33	45.41	0.243	0.785		0.104	0.902		1.060	0.589	
Ventral Anterior Insula - Rectus, R	62.43	49.62	71.47	51.55	86.01	54.05	0.976	0.382		1.344	0.267		3.016	0.221	
Ventral Anterior Insula - Ventral Striatum, R	571.32	83.02	592.98	51.51	606.57	49.91	0.678	0.511		2.005	0.142		0.391	0.142	
Medial OFC - BA10, L	30.29	0.00	30.29	0.00	30.29	0.00	100% of connectivity probability between regions and across groups								
Medial OFC - Hypothalamus, L	75.87	108.04	8.41	39.22	13.69	59.97	3.373	0.040	CW>AN*	6.470	0.003	CW>AN**; CW>BN*	29.385	<0.001	CW>AN***; CW>BN***
Medial OFC - Ventral Striatum, L	112.75	70.07	93.65	85.30	120.38	61.76	0.890	0.415		0.908	0.408		1.607	0.448	
Medial OFC - BA10, R	36.75	0.00	36.75	0.00	36.75	0.00	100% of connectivity probability between regions and across groups								
Medial OFC - Hypothalamus, R	16.88	21.33	3.82	10.83	81.89	322.12	1.032	0.362		1.309	0.276		9.335	0.009	CW>AN*; CW>BN*
Medial OFC - Ventral Striatum, R	87.45	95.24	149.96	108.23	159.66	92.04	3.549	0.034	AN>CW*; BN>CW*	4.050	0.021	BN>CW*	9.205	0.010	AN>CW*; BN>CW*
Middle OFC - BA10, L	25.55	0.00	25.55	0.00	25.55	0.00	100% of connectivity probability between regions and across groups								
Middle OFC - Hypothalamus, L	112.31	137.50	13.15	33.04	27.41	61.69	4.900	0.010	CW>AN**	9.316	<0.001	CW>AN***; CW>BN**	24.450	<0.001	CW>AN***; CW>BN***
Middle OFC - Ventral Striatum, L	130.10	50.88	141.63	67.83	177.60	41.36	2.522	0.088		5.217	0.008	BN>CW**	15.873	<0.001	AN>CW; BN>CW***
Middle OFC - BA10, R	29.75	0.00	28.61	5.84	29.75	0.00	1.787	0.175		0.980	0.380		1.962	0.375	
Middle OFC - Hypothalamus, R	107.32	152.40	9.61	20.10	17.11	65.06	3.997	0.023	CW>AN*	8.176	0.001	CW>AN***; CW>BN**	21.089	<0.001	CW>AN***; CW>BN***
Middle OFC - Ventral Striatum, R	111.39	68.57	105.43	93.87	143.88	97.24	1.040	0.359		1.422	0.248		2.604	0.272	
Rectus - BA10, L	27.04	7.30	17.76	13.37	16.39	11.97	6.722	0.002	CW>AN**; CW>BN**	6.928	0.002	CW>AN*; CW>BN**	9.773	0.008	CW>AN*; CW>BN*
Rectus - Hypothalamus, L	36.41	138.34	123.06	339.18	36.69	95.13	1.262	0.289		1.335	0.269		4.571	0.102	
Rectus - Ventral Striatum, L	99.36	81.38	187.61	84.77	220.88	58.42	3.071	0.053		17.532	0.000	AN>CW***; BN>CW***	24.595	<0.001	AN>CW**; BN>CW***
Rectus - BA10, R	24.11	6.47	21.18	9.85	26.30	7.24	2.898	0.062		2.642	0.078		5.507	0.064	
Rectus - Hypothalamus, R	16.45	62.39	56.76	132.11	132.01	364.30	1.368	0.261		1.739	0.183		2.257	0.323	
Rectus - Ventral Striatum, R	129.66	77.30	194.99	74.65	209.86	64.12	1.694	0.191		8.942	<0.001	AN>CW**; BN>CW***	15.203	<0.001	AN>CW**; BN>CW***
SN - Ventral Striatum, L	478.43	867.65	277.38	629.55	166.01	357.92	0.486	0.617		1.493	0.231		9.210	0.010	CW>BN*
SN - Ventral Striatum, R	87.09	119.46	128.30	426.48	289.28	1261.68	0.169	0.845		0.497	0.611		15.837	<0.001	CW>AN***; CW>BN**
VMP Thalamus - Frontal Inferior Operculum, L	177.69	319.88	101.87	150.65	501.63	1059.18	3.072	0.053		2.814	0.066		3.626	0.163	
VMP Thalamus - Dorsal Anterior Insula, L	1151.54	3801.86	1095.47	2531.04	1761.90	3164.49	0.270	0.764		0.336	0.716		12.547	0.002	BN>CW***
VMP Thalamus - Posterior Insula, L	4234.53	14355.41	6936.29	8634.12	9062.29	15510.83	0.763	0.470		0.865	0.425		26.138	<0.001	AN>CW***; BN>CW***
VMP Thalamus - Ventral Anterior Insula, L	121.99	243.22	1259.51	1877.18	992.21	2029.88	1.309	0.277		3.604	0.032	AN>CW*	21.159	<0.001	AN>CW***; BN>CW***
VMP Thalamus - Frontal Inferior Operculum, R	107.46	128.34	357.00	392.88	278.66	334.36	2.535	0.087		4.501	0.014	AN>CW*	12.450	0.002	AN>CW**; BN>CW**
VMP Thalamus - Dorsal Anterior Insula, R	398.66	427.99	1205.67	2885.69	1164.25	2176.02	0.574	0.566		1.211	0.304		8.355	0.015	BN>CW*
VMP Thalamus - Posterior Insula, R	1383.87	4502.88	20870.27	37368.60	11086.67	14463.96	1.883	0.160		4.517	0.014	AN>CW*	29.213	<0.001	AN>CW***; BN>CW***
VMP Thalamus - Ventral Anterior Insula, R	80.98	77.01	482.04	1149.47	1598.82	4404.09	0.727	0.487		2.329	0.105		5.314	0.070	

Supplemental Table 2. Probabilistic connectivity across groups analyzed using a) MANOVA with cofactors depression or anxiety diagnosis, SSRI or atypical antipsychotic use (Wilks' Lamba for Group Effect <0.001, p<0.001), b) MANOVA without cofactors (Wilks' Lamba for Group Effect was <0.001, p<0.001), as well as c) using a non-parametric test (Kruskal-Wallis with corrected pairwise post hoc comparison). Values are the same for all groups (SD 0.00) when connectivity probability was 100% within one or all groups, the path specific values vary based on correction of region size and path length. The shaded group contrasts indicate overlap for comparisons between the MANOVA with co factors and at least one of the other analysis methods.
